# Ankle joint mobility as a predictor of treatment prognosis in patients with chronic venous insufficiency with venous ulcers

**DOI:** 10.1590/1677-5449.180133

**Published:** 2019-06-25

**Authors:** Thiago Bertochi, Ricardo Zanetti Gomes, Mario Martins

**Affiliations:** 1 Hospital Universitário Regional dos Campos Gerais – HURCG, Cirurgia Geral, Ponta Grossa, PR, Brasil.

**Keywords:** arthrometry, articular, venous insufficiency, varicose ulcer

## Abstract

The present study arose from the need to improve treatment of patients with chronic venous insufficiency (CVI) who present with venous ulcers. A total of 40 lower limbs were assessed from 20 patients with healed venous ulcers (C5) or active venous ulcers (C6) who had undergone varicose vein surgery. The relationship between the range of motion of the ankle joint and presence of C5 or C6 venous ulcer was analyzed. For this purpose, normal goniometry findings for this joint were used as a predictor of venous ulcer healing, defined as the outcome. Thus, when identifying reduced ankle joint movement or immobility in these patients, new treatment options could be offered in order to increase joint mobility and prevent or delay CVI complications.

## INTRODUCTION

The prevalence of chronic venous insufficiency (CVI) is increasing, causing pain and incapacity and creating an important socioeconomic problem.^1^ If not properly treated, this condition can progress to its most severe form, active venous ulcers, leaving patients disabled and putting significant burden on the public purse.

The pathophysiology of venous ulcers is multifactorial,^2^ but ankylosis of the ankle is an important factor in the genesis of CVI, since if this joint becomes immobile, the ulcers can become incurable. However, in general, ankylosis is not a spontaneously occurring condition in these patients.^3^ Presence and degree of this ankylosis can be evaluated using a simple method: goniometry.

Correct calf muscle pump function helps in recovery from venous problems;^4^ and its restoration through physiotherapy, or even early recognition that it has become compromised, can prevent complications and minimize their signs and symptoms.^5^ There is a limited range of treatment options for patients with complications of CVI such as ulcers. Very often, physical recovery with physiotherapy after a surgical intervention is needed. However, many patients remain debilitated permanently because of the symptoms of CVI.^6^ Correctly prescribed elastic compression stockings are one of the most widely used treatments and they remain an important option for treatment of people with severe CVI.

The objective of this study is to assess goniometric measurement of the ankle joint as a predictive factor of treatment prognosis in patients with CVI and venous ulcers.

## METHODOLOGY

This is a cross-sectional, descriptive, and observational study conducted to evaluate venous ulcer healing progress during treatment of patients with compromised ankle joints. The following inclusion criteria were adopted: patients were recruited at the Lymphedema and Angiodysplasia Clinic, run by the Hospital Universitário Regional dos Campos Gerais, who had venous insufficiency classified as C5 or C6 on the CEAP classification (in which C = clinical, E = etiology, A = anatomic segment, and P = pathophysiology) and who may or may not have undergone surgical treatment for varicose veins. Patients were excluded if they had orthopedic, rheumatic, and/or neurological diseases involving the lower limbs or ulcers of other etiologies; if they had a history of lower limb fracture within the preceding 18 months; or if they refused to take part in the study.

**Figure 1:**
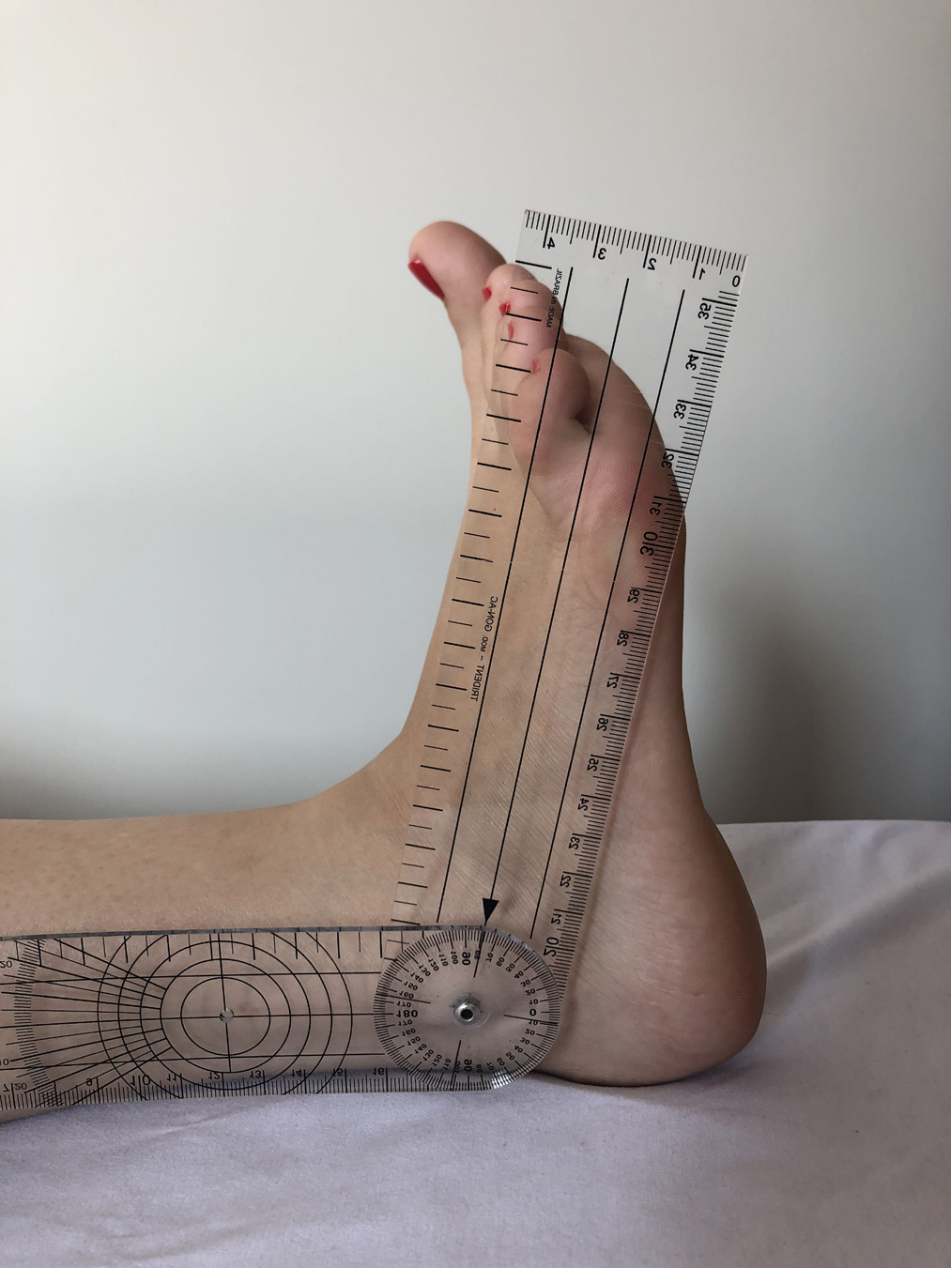
Goniometry of dorsiflexion.

**Figure 2:**
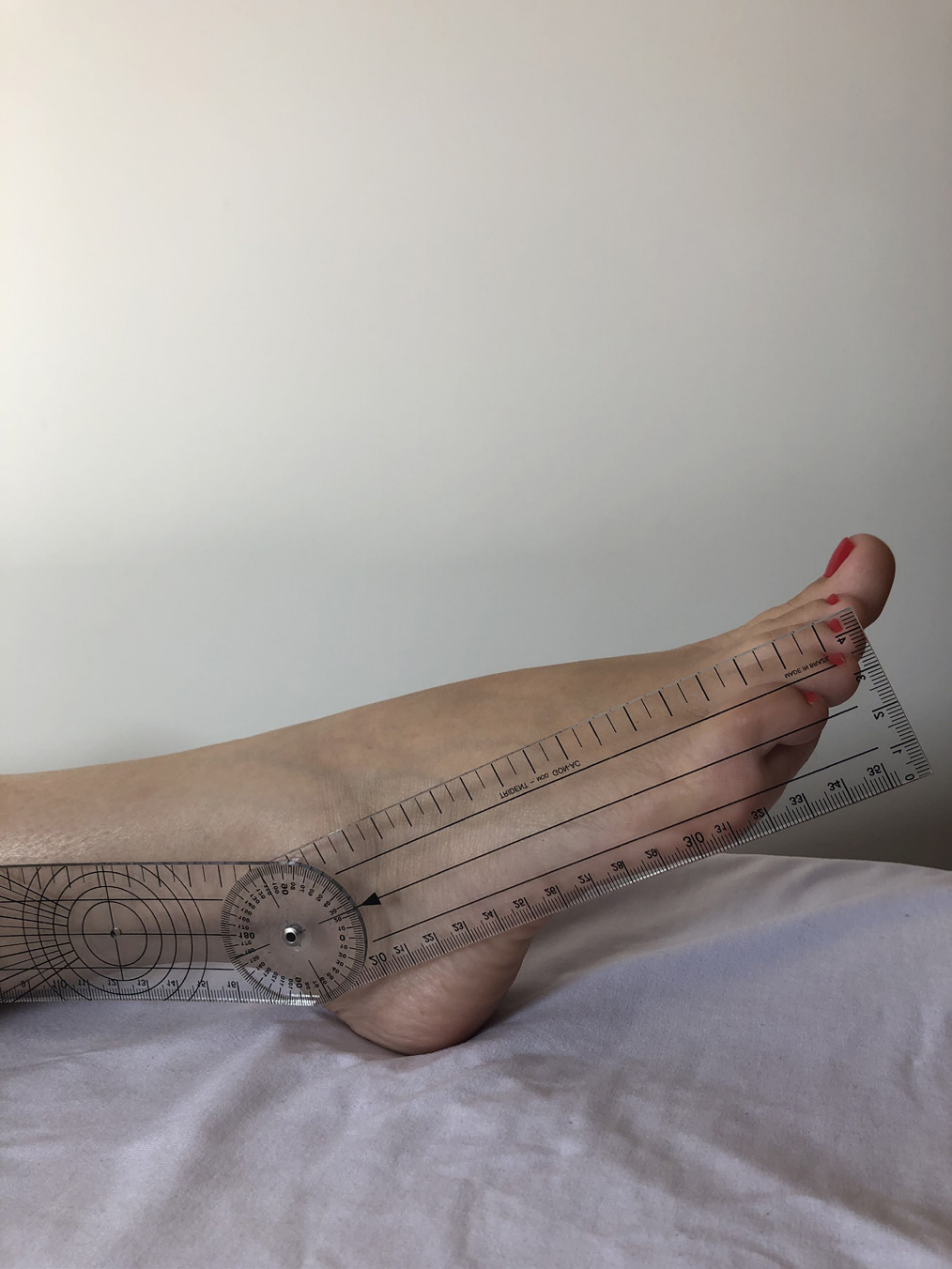
Goniometry of plantar flexion.

A total of 40 lower limbs were evaluated from 20 male and female patients who were being treated for CVI by the Lymphedema and Angiodysplasia Clinic at the Hospital Universitário Regional dos Campos Gerais. In accordance with the International Venous Disease Committee classification (known as the CEAP classification),^7^ limbs were assessed clinically and only those with a clinical classification of active venous ulcer (C6) or healed ulcer (C5) were selected. All participants were informed about the study objectives and signed a free and informed consent form that had been approved in advance by the Ethics Committee at the Universidade Estadual de Ponta Grossa. Patients were contacted by telephone and requested to return to the clinic at the Hospital Universitário Regional dos Campos Gerais in order to have plantar flexion and dorsiflexion amplitude measured. Measurements were all taken by the same researcher, using a universal goniometer, with the patient in decubitus dorsal, and taking one measurement per limb. The outcome of interest was complete healing or presence of active ulcer. In order to standardize the method,^8^ active dorsiflexion movements (Figure 1) and plantar flexion movements (Figure 2), were measured to determine the total travel, in the sagittal plane, between the distal extremities of the tibia and the fibula and the surface of the talus joint. Measurements of plantar flexion mobility were taken by placing the fixed arm of the goniometer parallel to the lateral surface of the fibula, the moving arm parallel to the lateral surface of the fifth metatarsal, and the angle over the ankle joint, against the lateral malleolus.^9^ Data were analyzed using IBM SPSS Statistics 2.0.^10^ The chi-square test was used to investigate whether there was a significant difference in the proportions of men and women whose ulcers had healed.

## RESULTS

A total of 40 lower limbs were analyzed from nine male patients and 11 female patients, with ages ranging from 49 to 84 years and a mean of 62.9 years. We observed that seven patients had undergone vascular surgery at times ranging from 40 days to 19 months prior to the assessments. It was also observed that 10 of the limbs analyzed exhibited dorsiflexion amplitude within normal limits and that 80% of these were free from venous ulcers. Overall, 10 limbs were free from ulcers, nine had healed ulcer (C5), and 21 had active ulcers (C6).

Tests were conducted to determine whether there were correlations between amplitude of ankle movement and healing. Irrespective of patient age or sex, limbs were classified into three groups (Table 1), where G1 had 0 to 10° of mobility, G2 had 11 to 20°, and G3 had greater than 20° dorsiflexion. Moderate to strong correlations were observed and feet with dorsiflexion in the range of 11 to 20° tended to heal better.

**Table 1:** Limbs classified into groups by amplitude of ankle joint movement, against the outcomes healed ulcer (C5), active ulcer (C6) or no ulcer.

**Ulcer/Movement**	**G1 (0-10°** * **)**	G2 (11-20°*)	G3 (> 20°*)
C5			
Dorsiflexion	1	7	1
Plantar flexion	2	3	4
C6			
Dorsiflexion	11	9	1
Plantar flexion	10	10	1
No ulcer			
Dorsiflexion	3	5	2
Plantar flexion	1	4	5

* Amplitude of ankle joint movement, measured using a universal goniometer.

Tests were also conducted for correlations between plantar flexion amplitude and healing. Irrespective of patient age or sex, limbs were classified into three groups (Table 1), where G1 had 0 to 10°, G2 had 11 to 20°, and G3 had greater than 20° of plantar flexion. No differences between groups were detected. Ulcer healing was equal for all degrees of plantar flexion. No significant difference was observed between groups (p > 0.05).

## DISCUSSION

This article describes an ongoing project that was initiated in response to a need to improve treatment for patients with CVI, with emphasis on secondary prevention. As such, statistical validation of the study results will be dependent on continuation of the research, in order to expand the patient sample observed.

Mobility of the ankle joint, in combination with competence of the calf muscle pump and preservation of venous system valve performance, is responsible for venous return to the heart.^11^ In the present study, compromise to this system was represented as reduced amplitude of ankle joint movement, or even total immobility, which constitutes an additional aggravating factor in lower limb CVI of venous ulcer patients. Therefore, people who exhibit reduced mobility in this joint will have worse prognosis for resolution of their ulcers, even after surgical treatment.^12^ For the purposes of this study, we considered normal variation of movement to be 45° for plantar flexion and 20° for dorsiflexion.^13^ Statistically, there is no difference in ankle mobility between the left and right sides.^14^ We did not therefore make any distinction between the side assessed.

Ankle joint immobility can suppress calf muscle pump activity and is one of the causes of untreatability among patients with venous ulcers,^15^ because, when walking, the deep layer of the crural fascia triggers contraction of the triceps-surae, which only functions adequately if amplitude is preserved.^16^ The association between reduced ankle joint mobility and venous ulcers was described for the first time in 1931.^17^ More recently, in 1982, it was observed that 32% of patients with advanced venous lesions had some degree of ankle joint immobility.^18^ However, in the present study it was observed that a favorable outcome was more common among patients with dorsiflexion in the range of 11 to 20° than those with mobility exceeding 20°.

No differences associated with plantar mobility were detected between the groups analyzed.

We emphasize that loss of ankle mobility precedes the appearance of ulcers in patients with CVI and, as the lesions worsen, there is concomitant reduction in the amplitude of mobility.^12^ However, we observed that the degree of mobility was not important for resolution of ulcers.

Thus, patients with longstanding C6 disease have greater rigidity than those whose ulcers respond favorably who, in turn, have greater rigidity than those who have never had ulcers. Thus, if appropriate exercises are capable of improving physiological venous return function, they may also offer additional therapeutic benefits.^19^ This study, therefore, starts from the assumption that vascular physiotherapy for CVI can prevent lesions from worsening, promoting rehabilitation and aiding treatment of this vascular condition. This would prevent the patient from suffering functional losses, minimizing the clinical consequences of the disease and contributing to healing of the venous ulcer. Therefore, although the results of this study did not correlate with published data, because of the small sample, it is nevertheless suggested that ankle goniometry should be adopted as a routine part of physical examination of these patients, in order to achieve adequate prevention and treatment planning. Furthermore, in view of the importance of the subject, we aim to continue the study, with additional data collection, in order to increase the number of patients analyzed with a view to attaining statistical relevance and achieving correspondence between the data observed and published data.

## CONCLUSIONS

The data observed do not correspond with those described in the literature, because of the small sample. Nevertheless, on the basis of information obtained from the bibliography consulted, we conclude that preservation of ankle joint mobility offers benefits for primary, secondary, and tertiary prevention of the complications of CVI. Routine assessment of goniometry at initial consultations with CVI patients and during ongoing follow-up offers information that is important when deciding on the best treatment for these patients. New treatment modalities could be proposed with the objective of provoking increased ankle mobility through exercise, in order to prevent or delay the complications of CVI and also to predict the success of surgical treatment for patients with this condition. In view of the importance of the subject, we aim to continue the study, increasing the size of the sample, in order to achieve statistical relevance.
