# Supplementary material for: Ankle joint mobility as a predictor of treatment prognosis in patients with chronic venous insufficiency with venous ulcers
Source: J Vasc Bras. 2019 Jun 25;18:e20180133. doi: 10.1590/1677-5449.180133 (PMC6636814; doi:10.1590/1677-5449.180133)
Supplement: Translation

# Translation: Mobilidade da articulação talocrural como fator preditor no prognóstico de cicatrização em portadores de insuficiência venosa crônica com úlcera venosa

**Authors:** Thiago Bertochi, Ricardo Zanetti Gomes, Mario Martins

**Affiliations:** 1 Hospital Universitário Regional dos Campos Gerais – HURCG, Cirurgia Geral, Ponta Grossa, PR, Brasil.

**Keywords:** artrometria articular, insuficiência venosa, úlcera varicosa

## Abstract

O presente trabalho surgiu da necessidade de aprimorar o tratamento de pacientes com insuficiência venosa crônica (IVC) que apresentam úlcera venosa. Foram avaliados os 40 membros inferiores de 20 pacientes portadores de úlcera venosa cicatrizada (C5) ou ativa (C6) que foram submetidos ou não à cirurgia de varizes. Foi estabelecida a relação entre a amplitude de movimento da articulação talocrural e a presença de úlcera venosa C5 ou C6, sendo para isso utilizada a goniometria dessa articulação dentro da normalidade como preditor de cicatrização e tendo como desfecho a cicatrização ou não da úlcera venosa. Dessa forma, ao se identificar redução ou imobilidade talocrural em pacientes com úlcera venosa, poderão ser oferecidas novas formas terapêuticas, a fim de aumentar a mobilidade dessa articulação para prevenir ou retardar complicações da IVC.

## INTRODUÇÃO

A prevalência da insuficiência venosa crônica (IVC) está aumentando, causando dor e incapacidade e gerando um importante problema socioeconômico^1^. Caso não se realize tratamento adequado, essa enfermidade pode evoluir para sua forma mais grave, a úlcera venosa ativa, levando o indivíduo à invalidez e elevando os gastos dos cofres públicos.

A fisiopatologia da úlcera venosa é multifatorial^2^, porém a anquilose do tornozelo é um importante fator na gênese da IVC, uma vez que, ao se reconhecer a imobilidade dessa articulação, as lesões podem se tornar incuráveis. Contudo, em geral, a anquilose não é uma queixa espontânea nesses pacientes^3^. Para analisar a ocorrência dessa condição, a flexibilidade das articulações pode ser avaliada através de um método simples, a goniometria.

O adequado funcionamento da bomba muscular da panturrilha auxilia na recuperação dos problemas venosos^4^; portanto, sua restauração através de fisioterapia, ou mesmo o reconhecimento precoce do seu comprometimento, pode prevenir complicações, além de minimizar seus sinais e sintomas^5^. As opções de tratamento para pacientes com complicações da IVC, como a úlcera, são limitadas. Muitas vezes é necessária uma recuperação física com fisioterapia após a intervenção cirúrgica. No entanto, muitos pacientes permanecem cronicamente debilitados como resultado dos sintomas da IVC^6^. Meias elásticas compressoras adequadamente prescritas, uma das principais terapias utilizadas, continuam sendo importantes no tratamento de indivíduos com IVC severa.

O objetivo do presente trabalho é avaliar a mensuração goniométrica da articulação tíbio-társica como fator preditor no prognóstico do tratamento em portadores de IVC com úlcera venosa.

## METODOLOGIA

Trata-se de um estudo de corte transversal, descritivo e observacional para avaliar a evolução da cicatrização da úlcera venosa no tratamento de indivíduos com comprometimento da articulação talocrural. Foram adotados os seguintes critérios de inclusão: pacientes oriundos do ambulatório de cirurgia vascular do Hospital Universitário Regional dos Campos Gerais e pacientes com insuficiência venosa classe C5 e C6 da classificação CEAP (na qual C = clínica, E = etiologia, A = segmento anatômico e P = fisiopatologia) que foram submetidos ou não ao tratamento cirúrgico de varizes. Foram excluídos indivíduos portadores de doenças ortopédicas, reumatológicas e/ou neurológicas de membros inferiores ou de úlceras de outras etiologias; indivíduos com história de fraturas em membros inferiores há 18 meses; e os que se recusaram a participar do estudo.

Foram então avaliados os 40 membros inferiores de 20 pacientes masculinos e femininos, que estavam em acompanhamento no ambulatório de cirurgia vascular do Hospital Universitário Regional dos Campos Gerais por IVC. De acordo com a classificação do Comitê Internacional em Doença Venosa, denominada classificação CEAP^7^, os membros foram clinicamente avaliados e selecionados exclusivamente conforme a classificação clínica de úlcera venosa ativa (C6) e úlcera cicatrizada (C5). Todos os participantes assinaram um termo de consentimento livre e esclarecido após serem informados sobre o objetivo deste estudo, que foi previamente aprovado pelo Comitê de Ética da Universidade Estadual de Ponta Grossa. Foi então realizada uma convocação mediante contato telefônico, para retornarem ao ambulatório do Hospital Universitário Regional dos Campos Gerais a fim de se submeterem à medida de amplitude da flexão plantar e dorsal, com o auxílio de goniômetro universal, executada em posição de decúbito dorsal, sempre pelo mesmo pesquisador, em uma única medida para cada membro. Os desfechos de interesse foram: cicatrização completa ou presença de úlcera ativa. Para padronização do método^8^, tratou-se de avaliar os movimentos ativos de flexão dorsal (Figura 1) e plantar (Figura 2), para mensuração da cinesia, no plano sagital, entre as extremidades distais da tíbia e da fíbula e a superfície articular do tálus. Para a mensuração da mobilidade de flexão plantar, foi posicionado o braço fixo do goniômetro paralelo à face lateral da fíbula, o braço móvel paralelo à superfície lateral do quinto metatarso e o eixo sobre a articulação do tornozelo, junto ao maléolo lateral^9^. A análise dos dados foi realizada com auxílio do programa SPSS IBM Statistic 2.0^10^. Foi aplicado o teste de qui-quadrado para investigar se existia diferença significativa na cicatrização das feridas entre homens e mulheres.

**Figura 1:** Goniometria da flexão dorsal.

**Figura 2:** Goniometria da flexão plantar.

## RESULTADOS

Foram analisados os 40 membros inferiores de nove pacientes do sexo masculino e 11 do sexo feminino, com idade entre 49 e 84 anos, média de 62,9 anos. Observamos que sete pacientes foram submetidos a cirurgia vascular num período de 40 dias a 19 meses antes da avaliação. Foi observado ainda que 10 membros analisados apresentaram amplitude de dorsiflexão dentro da normalidade e, destes, 80% eram isentos de lesão venosa, perfazendo um total de 10 membros sem lesão, nove com úlcera cicatrizada (C5) e 21 com úlcera ativa (C6).

Para avaliação da correlação da amplitude de movimento talocrural com a cicatrização, o teste de correlação foi aplicado. As lesões, independentemente da idade ou do sexo, foram divididas em três grupos (Tabela 1), sendo G1 entre 0 e 10°, G2 entre 11 e 20° e G3 acima de 20° de dorsiflexão. Foi observada correlação moderada para alta, sendo que lesões em pés com dorsiflexão entre 11 e 20° tenderam a cicatrizar melhor.

**Tabela 1:** Divisão dos membros avaliados em grupos de acordo com a amplitude de movimento da articulação talocrural em relação à presença de úlcera cicatrizada (C5), ativa (C6) ou ausência de úlcera.

**Úlcera/Movimento**	**G1 (0-10°** * **)**	G2 (11-20°*)	G3 (> 20°*)
C5			
Dorsiflexão	1	7	1
Flexão plantar	2	3	4
C6			
Dorsiflexão	11	9	1
Flexão plantar	10	10	1
Sem úlcera			
Dorsiflexão	3	5	2
Flexão plantar	1	4	5

* Amplitude de movimento da articulação talocrural, mensurado mediante uso do goniômetro universal.

Para avaliação da correlação da amplitude de flexão plantar com a cicatrização, o teste de correlação foi aplicado. As lesões, independentemente da idade ou do sexo, foram divididas em três grupos (Tabela 1), sendo G1 entre 0 e 10°, G2 entre 11 e 20° e G3 acima de 20° de flexão plantar. Não foi observada nenhuma diferença entre os grupos. A resolução da ferida foi igual em qualquer grau de flexão plantar. Não foi observada diferença significativa entre os grupos (p > 0,05).

## DISCUSSÃO

O presente estudo refere-se a um projeto em desenvolvimento, que surgiu a partir da necessidade de aprimorar o tratamento dos pacientes com IVC, dando ênfase na prevenção secundária desta. Portanto, a validação estatística dos resultados deste estudo irá depender do prosseguimento deste estudo, a fim de ampliar o universo de pacientes observados.

A mobilidade da articulação talocrural, associada à competência da bomba muscular da panturrilha e à preservação do desempenho do sistema valvular venoso, proporciona o retorno venoso de volta ao coração^11^. O comprometimento desse sistema, representado neste estudo pela amplitude de movimento talocrural reduzida ou mesmo pela completa imobilidade, estabelece um fator agravante da IVC dos membros inferiores em portadores de úlcera venosa. Portanto, indivíduos que apresentam comprometimento da mobilidade dessa articulação irão apresentar um pior prognóstico na resolução da lesão ulcerosa, apesar do tratamento cirúrgico^12^. Consideramos, para este trabalho, uma variação normal de movimento: 45° para flexão plantar e 20° para dorsal^13^. Estatisticamente não há diferença na mobilidade do tornozelo entre os lados esquerdos e direito^14^. Portanto, em nossa pesquisa, não fizemos distinção entre o lado avaliado.

A imobilidade articular talocrural pode suprimir a ação da bomba muscular da panturrilha, sendo uma das causas de intratabilidade de pacientes com úlcera venosa^15^, pois a camada profunda da fáscia crural, durante a marcha, promove a contração do músculo tríceps-sural, cuja ação só se realiza adequadamente mediante amplitude preservada^16^. Essa associação entre redução da mobilidade da articulação talocrural e úlceras venosas foi descrita pela primeira vez^17^ em 1931. Mais tarde, em 1982, foi observado que 32% dos pacientes com lesões venosas avançadas apresentavam algum grau de imobilidade da articulação do tornozelo^18^. Por outro lado, no presente estudo foi observado que um desfecho favorável se encontra naqueles pacientes com uma dorsiflexão entre 11 e 20°, em comparação àqueles com mobilidade acima de 20°.

Já em relação à mobilidade plantar, não foi encontrada diferença entre os grupos analisados.

Ressaltamos que a perda de mobilidade talocrural antecede o aparecimento da úlcera naqueles indivíduos portadores de IVC e, conforme se agravam as lesões, ocorre redução concomitante da cinesia em estudo^12^. No entanto, observamos que o grau de mobilidade foi indiferente para resolução da ferida.

Dessa forma, aqueles com insuficiência C6 há longo tempo apresentam maior enrijecimento em relação àqueles com evolução favorável das lesões; e estes, maior enrijecimento em relação aos sem úlceras prévias. Então, se exercícios apropriados têm a capacidade de aumentar a função fisiológica do retorno venoso, podem também apresentar benefícios terapêuticos adicionais^19^. Este trabalho, portanto, parte do pressuposto de que a fisioterapia vascular na IVC pode prevenir o agravamento das lesões, promovendo a reabilitação e auxiliando no tratamento dessa alteração vascular. Dessa forma, evita-se a perda funcional do indivíduo, minimizando as consequências clínicas da doença e auxiliando na cicatrização da úlcera venosa. Portanto, apesar de o presente estudo não ter tido correlação com dados da literatura, devido à amostra reduzida, sugere-se que a goniometria talocrural seja introduzida de maneira rotineira durante o exame físico desses pacientes, a fim de buscar a prevenção e planejamento da terapia adequada. Contudo, diante da relevância do tema, objetivamos o prosseguimento da pesquisa, com aumento de coleta de dados, a fim de estender a quantidade de pacientes analisados, para alcançar importância estatística, com o objetivo de encontrar correspondência dos dados da literatura com os dados obtidos.

## CONCLUSÃO

Não foi obtida correspondência dos dados encontrados com os descritos na literatura, devido à amostra reduzida. Contudo, através de informações levantadas com base na bibliografia utilizada, concluímos que a preservação da mobilidade da articulação talocrural traz benefícios na prevenção primária, secundária e terciária das complicações da IVC. Dessa forma, a avaliação, de maneira rotineira, da goniometria em consulta inicial de portadores IVC e em sua evolução, apresenta uma importante informação no momento de optar pelo melhor tratamento desses pacientes. Podem ser propostas novas formas de terapêutica com o intuito de promover o aumento da mobilidade talocrural através de exercícios, a fim de prevenir ou retardar complicações da IVC e ainda predizer o sucesso do tratamento cirúrgico de pacientes com essa condição. Devido à relevância do tema, propomos o prosseguimento deste estudo com aumento da amostra, para alcançar uma significância estatística nos dados encontrados.
